# DNA unchained: two assays to discover and study inhibitors of the DNA clustering function of barrier-to-autointegration factor

**DOI:** 10.1038/s41598-020-69246-x

**Published:** 2020-07-23

**Authors:** Michael Burger, Caroline Schmitt-Koopmann, Jean-Christophe Leroux

**Affiliations:** Institute of Pharmaceutical Sciences, ETH Zurich, Zurich, Switzerland

**Keywords:** Drug delivery, Gene delivery, Transfection, Gene therapy, Drug discovery, High-throughput screening

## Abstract

The protein barrier-to-autointegration factor (BAF) and its interaction partners, the LEM (LAP2B, emerin, MAN1)-domain proteins, constitute a powerful cytoplasmic DNA defense mechanism. Invading DNA molecules are quickly bound by the BAF system and trapped in membrane compartments. This decreases the nuclear uptake of DNA from the cytoplasm. Inhibition of the BAF system is therefore expected to enhance the efficacy of non-viral DNA transfection agents. In this study, we introduced a protocol for the recombinant expression of soluble BAF and developed two ELISA-type assays to discover small molecule inhibitors of BAF-dependent DNA retention by high throughput screening (HTS). The proton pump inhibitor rabeprazole as well as three compounds of the Maybridge library were identified as inhibitors of the LEM-BAF-DNA interaction chain. The inhibition was based on adduct formation with BAF cysteine residues. An enhancing effect of the compounds on cell culture transfection, however, was not observed, which may be attributed to the reducing environment of the cytoplasm that prevents the adduct formation with BAF cysteine residues. The novel assays developed here can provide new tools to further study the biological functions of the BAF system, and may lead to the identification of suitable BAF inhibitors in future HTS campaigns.

## Introduction

The non-viral delivery of double stranded DNA (dsDNA) into the nucleus of mammalian cells is a notoriously difficult undertaking. Passive uptake routes are not applicable for such large and highly charged molecules, and their endocytosis is often so inefficient that most of the administered payload is lost due to extracellular degradation and/or systemic clearance^1,2^. The small fraction of DNA that is taken up by the target cells must then escape quickly from the endosomal compartments to avoid hydrolysis in the lysosomes, be transported through the highly crowded cytoplasmic environment^3^, and finally be smuggled into the nucleus^4,5^.

These hurdles are well known and delivery efforts in the field of gene therapy have focused on developing new complexing agents or modifying the existing ones, to increase serum stability, uptake, selectivity and endosomal escape of DNA^6,7^. The efficacy of gene delivery systems has indeed improved significantly over the last decade, yet the translation into reliable in vivo applications remains slow. New paths should be explored to accelerate the development of non-viral gene therapy towards clinical application. One possible strategy, which to our knowledge has only been minimally explored, is the coadministration of the complexed DNA with small molecule transfection enhancers^8,9^. In this study, we developed two ELISA-type assays to find inhibitors of a powerful cellular defense system against dsDNA, which is constituted by the protein barrier-to-autointegration factor (BAF) and its interaction partners, the LEM-domain proteins. In the cytoplasm, the BAF system is likely responsible for a considerable reduction of the mobility of exogenous DNA and prevents its efficient nuclear uptake.

BAF is a 10 kDa, ubiquitously expressed protein that is prevalent mostly as a homodimer. Each monomer contains a sequence non-specific DNA binding site^10–12^. In the homodimerized state the DNA binding affinity is in the low nanomolar range^12^, and the DNA binding sites are oriented towards opposite sides of the complex, allowing BAF to effectively establish intermolecular DNA bridges (Supplementary Fig. S1)^10,11^ and hence organize the DNA in a mesh-like cluster^13^. In interphase, BAF is mostly found in the nucleus, with the highest concentration at the interface between the chromatin and the inner nuclear membrane. There, it binds to DNA as well as certain DNA histones and simultaneously to LEM-domain proteins, such as emerin in the inner nuclear membrane^14,15^. Entering mitosis, the DNA binding activity of BAF is inhibited through hyperphosphorylation by the vaccinia related kinases (VRK)^16^. Consequently, BAF detaches from the chromatin and becomes dispersed in the cytoplasm as the nucleus disassembles until it is again dephosphorylated in telophase by the protein phosphatase 2 (PP2A). Once dephosphorylated, BAF accumulates at the heterochromatic regions of the segregated chromosomes and recruits—via the interaction with the LEM-domain proteins—endoplasmatic reticulum (ER) membranes to cover the genomic DNA. This process eventually leads to the reassembly of the nuclear envelope, which makes BAF an indispensable player for the survival of dividing cells^17^.

**Figure 1:**
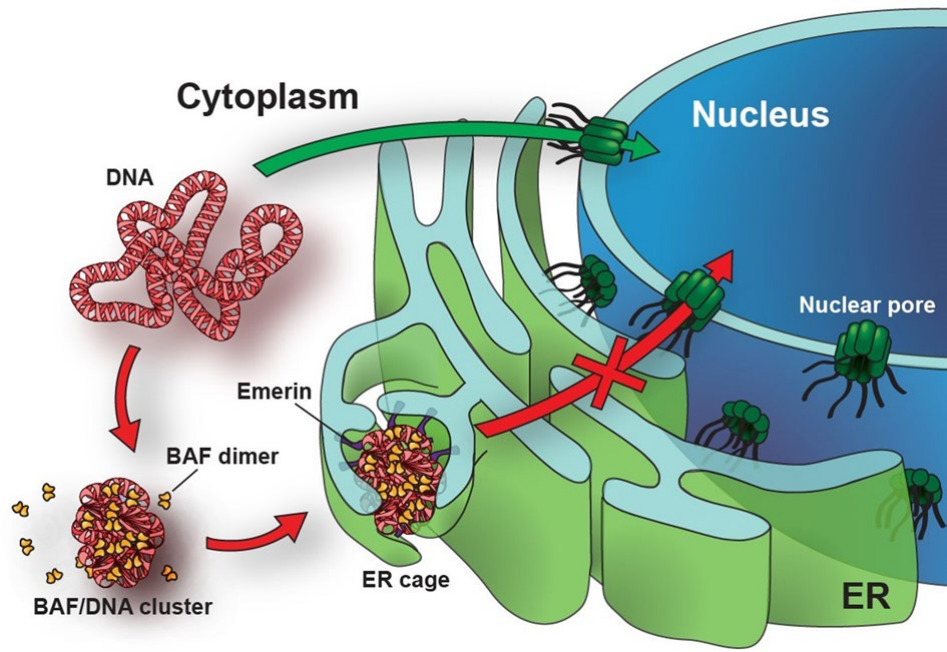
LEM-BAF mediated cytoplasmic DNA retention. Schematic representation of the cytoplasmic DNA clustering by BAF and emerin. Cytoplasmic DNA (red) is bound and clustered by the BAF protein (yellow). BAF recruits emerin and other ER resident LEM-domain proteins, which leads to the retention of the DNA in membrane cages.

From a gene delivery perspective, it is unfortunate that dephosphorylated BAF is also present in the cytoplasm of interphase cells and that LEM-domain proteins cover the ER membranes. As a consequence, exogenous DNA that enters the cytoplasm is quickly bound and clustered by BAF, and soon entrapped in membrane cages^18–22^. This mechanism of DNA retention manifests as DNA foci in the cytoplasmic region (Fig. 1), which appear only hours after transfection and remain relatively immobile over several days, even throughout mitosis^23^. The fate of the entrapped DNA is not yet understood but it can be hypothesized that it fails to be translocated to the nucleus.

Interestingly, viruses, which unpack and amplify their DNA genome in the cytoplasm, express a VRK homologue to protect their genome from BAF induced DNA aggregation. For instance, vaccinia virus depends on the activity of its B1 kinase to protect its genome from BAF activity, and clearly suffered a decrease in transduction efficacy in the absence of the kinase^24^. On the other hand, BAF can also have proviral functions. It was shown to play a role in the formation of the preintegration complex in retroviruses, and prevents the occurrence of genomic autointegration^25,26^. Other cellular functions were also reported to involve BAF such as its effect on DNA repair and genome stability through the regulation of the poly [ADP-ribose] polymerase 1 (PARP1) activity^27^.

Only one compound, obtusilactone B, was so far described to directly bind BAF^28^. However, obtusilactone B does not affect DNA clustering or the interaction with the LEM-domain proteins. It rather prevents the inactivation of BAF by blocking its interaction with vaccinia-related kinase 1 (VRK1). Furthermore, several inhibitors of the BAF regulating enzymes VRK1 and PP2A have been described^29–33^. Those compounds are mainly characterized as cytotoxic agents and were considered as anti-cancer treatments.

We hypothesized that a small molecule inhibitor of the LEM-BAF-DNA interaction chain would improve the transfection efficiency of dsDNA by preventing it from clustering and/or being entrapped in membrane cages. To investigate this, we introduced a protocol for the recombinant expression of soluble BAF and developed two ELISA-type assays for high throughput screening (HTS) of small molecule libraries to identify potential inhibitors of the BAF-dependent DNA clustering. The herein described assays can be further used to study other biological functions of the BAF system and monitor the activities of BAF regulating factors of mammalian, bacterial and viral origin. This is illustrated in the present study on the example of the human kinase VRK1.

## Results

### Screening assays development

**Figure 2:**
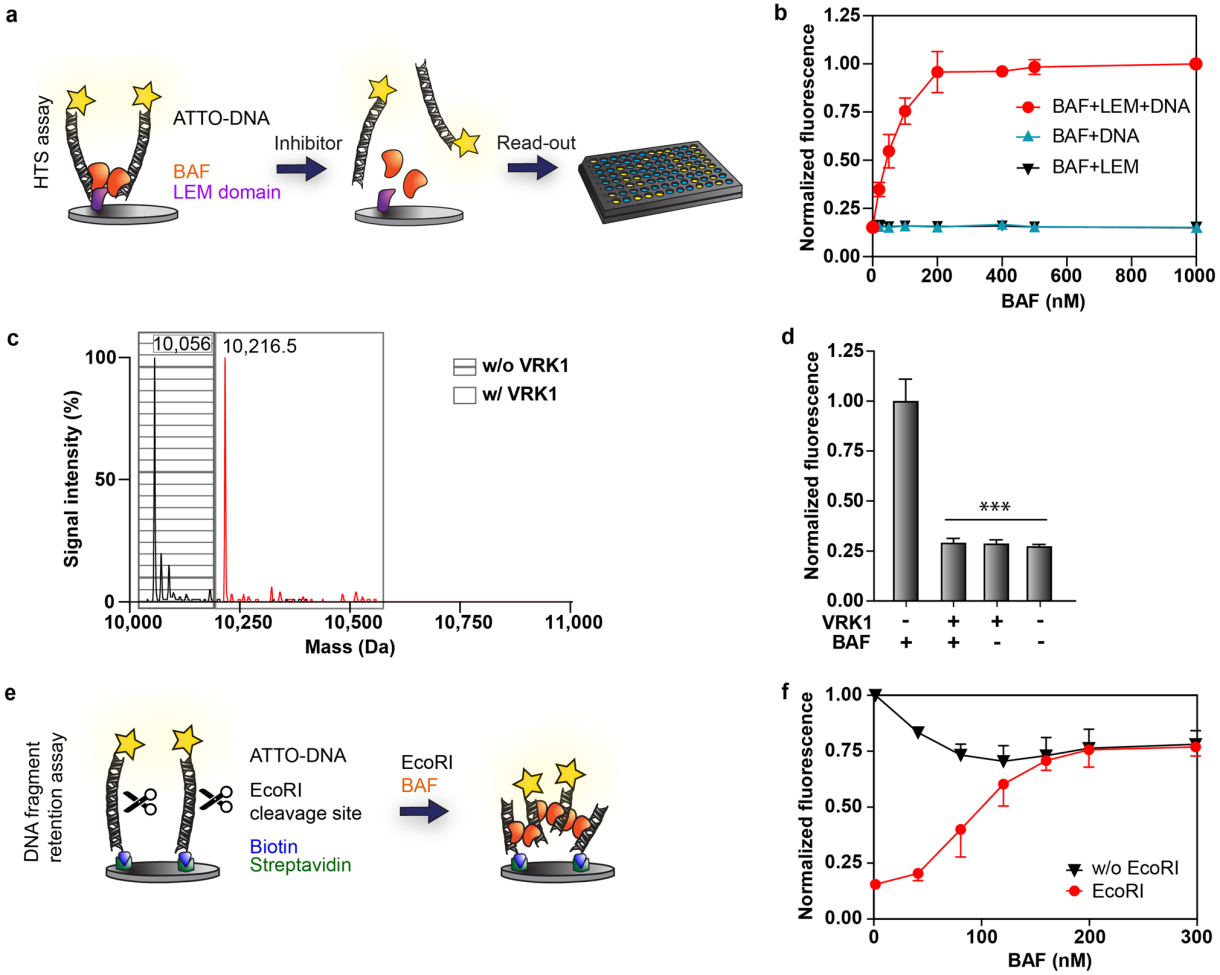
The setup of the two screening assays. (**a**) Schematic representation of the HTS assay. The LEM-domain of human emerin is immobilized on the bottom of a multiwell plate. In the presence of BAF, the ATTO 425-labelled polymerase chain reaction (PCR) product is retained in the well or washed away if any part in the LEM-BAF-DNA chain is missing. (**b**) The HTS assay was performed with increasing concentrations of BAF (circles), without the LEM-domain (up triangles) or without DNA (down triangles). Fluorescence was normalized with respect to the BAF (1000 nM) value (BAF + LEM + DNA). Mean ± SD (N = 4). (**c**) ESI–MS analysis of BAF (10,056 Da) without (black peaks in dashed box) and with preincubation with human VRK1 (red peaks in clear box). (**d**) The HTS assay was conducted with or without VRK1 pre-treated BAF. Fluorescence was normalized and statistical significance tested with respect to the sample without VRK1 pre-treatment. Mean + SD (N = 3). ***p < 0.01. (**e**) Schematic representation of the DNA fragment retention (DFR) assay. A 270-bp ATTO 425-labelled and biotinylated PCR product is immobilized on a streptavidin coated multiwell plate. Upon the addition of BAF and a restriction enzyme (here EcoRI), the fluorescently labelled DNA terminus is cleaved off and is only retained in the well due to BAF clustering. (**f**) The DFR assay was performed with increasing BAF concentrations, both in the presence (circles) or absence (triangles) of EcoRI. Fluorescence was normalized with respect to the BAF (0 nM) value w/o EcoRI. Mean ± SD (N = 3).

Two ELISA-type assays were developed to screen for small molecule effectors of BAF. The first assay involves the LEM-BAF-DNA interaction chain, and was set up for HTS on a robotic platform. In this assay, the LEM-domain of human emerin is immobilized on the bottom of the well, thereby mimicking the situation on the ER membranes, while BAF and 270 bp ATTO 425-labelled DNA are added in soluble form (Fig. 2a)**.** When the LEM-BAF-DNA interaction chain is intact, the fluorescently labelled DNA is clustered by BAF and retained on the LEM-coated surface, which results in a high fluorescence signal. It was found that maximal fluorescence signal, and thus DNA retention, was achieved at a BAF concentration of 200 nM or higher (Fig. 2b: circles). On the other hand, if any part of the interaction chain is missing, the DNA is washed away and the fluorescence signal diminishes (Fig. 2b: triangles). In the assay setup, the non-DNA binding BAF mutant BAF G27E^34^ was used to simulate complete BAF inactivation. However, to increase the assay throughput this control was later changed to omitting BAF from the control wells. Both controls resulted in undetectable DNA retention (Supplementary Fig. S2).

The HTS assay was challenged under various conditions, such as the presence of heparan sulfate, RNA, DNA and a wide range of pH and salt concentrations (Supplementary Fig. S3). The retention of fluorescently labelled DNA was not affected by either heparan sulfate or moderate concentrations of RNA. However, the addition of unlabelled plasmid DNA strongly competed for BAF binding with the labelled DNA probe and reduced the signal intensity. Moreover, the assay was stable under a wide range of salt concentrations (up to 1 M) and pH values (pH 5–8). Aside from screening compound libraries, this assay can also be utilized to study BAF regulating enzymes, which is demonstrated here for the example of the human kinase VRK1. When BAF was preincubated with VRK1, phosphorylation occurred at 2 residues, as indicated by a 160 Da mass shift from 10,056 Da to 10,216.5 Da (Fig. 2c). The phosphorylated BAF was inactive in the HTS assay (Fig. 2d), since its DNA binding affinity to DNA was impaired^35^.

The secondary assay, *i.e.* the DNA fragment retention (DFR) assay, was developed to test the HTS hits under conditions that only involve the interaction between BAF and DNA, but not the LEM-domain. This allows to differentiate inhibitors of the BAF-DNA interaction (active in both assays) from those that interfere with the binding of BAF to the LEM-domain (active only in the HTS assay). In the DFR assay, linear pieces of DNA with a single EcoRI or HindIII cleavage site in the middle (Supplementary Fig. S4) were generated, which were biotinylated on one end and fluorescently labelled on the other. A streptavidin coated multiwell plate was coated with the DNA molecules, and BAF was added together with the restriction enzyme of choice, e.g., EcoRI (Fig. 2e).

The restriction enzyme cleaves the DNA into two fragments: a biotinylated fragment that remains immobilized on the bottom of the well and a labelled fragment that is now solubilized. In the absence of BAF, the detached DNA fragments are removed in the washing steps, resulting in a diminished fluorescence signal. In the presence of BAF on the other hand, the fluorescently labelled DNA fragments are clustered with the immobilized fragments, preventing them from being washed away and leading to increasing fluorescence signals with increasing BAF concentrations (Fig. 2f, circles). Similarly to the HTS assay, maximal DNA retention was achieved at a BAF concentration of 200 nM.

In the absence of restriction enzyme, a reduction of the fluorescence signal can be observed with increasing BAF concentration (Fig. 2f: triangles). This could be attributed to fluorescence quenching as a result of BAF-dependent DNA clustering. Importantly, we showed that the restriction enzyme was still able to hydrolyze the DNA in the presence of BAF (Supplementary Fig. S5), and that different restriction enzymes can be applied as long as the respective cleavage site is included in the DNA sequence (Supplementary Fig. S6). The latter is the case for EcoRI and HindIII but not for the enzyme XhoI. Accordingly, XhoI did not lead to fragmentation of the immobilized DNA molecule.

### Screening results

The HTS assay was first used to screen manually the Pfizer licensed compound library at a concentration of 10 μM in triplicates (Table 1). Out of 94 compounds, only one hit was identified. This was the cytotoxic drug doxorubicin, which reduced the fluorescence intensity by about 50%, whereas the remaining compounds did not lead to a significant decrease in fluorescence intensity.

**Table 1:** Summary of pilot screening results obtained with the HTS assay. Mean Z' factor ± SD.

Library Name	Number of compounds	Mean Z’ factor	Total hits	False positives	DNA intercalators	True positives
Pfizer licensed compound library (manual)	94	0.82 ± 0.08	1	0	Doxorubicin	0
Prestwick chemical library	1273	0.58 ± 0.09	41	36	Doxorubicin Epirubicin Propidium iodide Daunorubicin	Rabeprazole
Protein–Protein interaction library	5398	0.36 ± 0.09	48	48	0	0
Chemical diversity library	7649 (50,000)^a^	0.44 ± 0.08	82	82	0	0
Natural product collection	2603	0.36 ± 0.14	26	26	0	0
Maybridge library	13,974	0.33 ± 0.13	65	62	0	KM 04550 KM 04416 JFD 02731

^a^7649 compounds were screened, representing the chemical diversity of the whole library (50,000 compounds).

The assay protocol was thoroughly optimized in order to repeatedly achieve a Z’ factor greater than 0.5 with the robotic platform (Supplementary Fig. S7)^36^. Subsequently, the Prestwick chemical library, the protein–protein interaction library, the chemical diversity library (7649 compounds, representing the structural diversity of the whole 50,000 compound collection), the natural product collection and the Maybridge library were screened (a total of ~ 31,000 compounds) with the robotic platform at a concentration of 10 µM in duplicates (Table 1). This resulted in 262 primary hits, which were then categorized in three groups. The first and largest group constituted false positive hits, which could not be confirmed by manual repetition of the HTS assay in triplicates. The second group were known DNA intercalators, such as doxorubicin, that robustly decreased the signal intensity. However, the DNA intercalators could not be removed from the DNA in the washing steps and quenched the ATTO 425 fluorescence signal (Supplementary Fig. S8). This class of molecules was therefore excluded from subsequent screenings and not considered further. The third group contained four compounds that were confirmed as hits by manual repetition of the HTS assay and did not fall under the category of DNA intercalators, thereby being classified as true positive hits.

#### Rabeprazole

**Figure 3:**
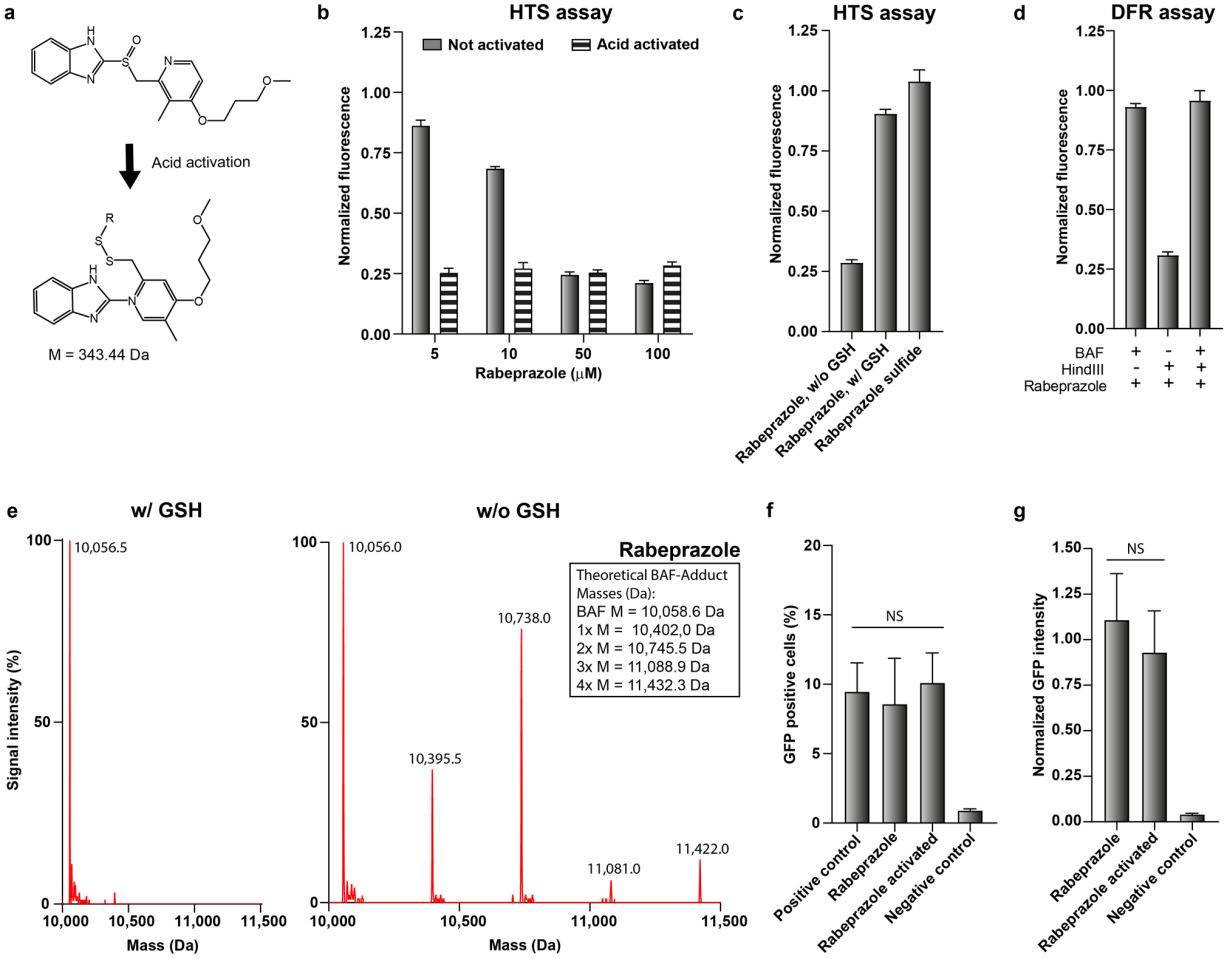
Validation of rabeprazole as LEM-BAF inhibitor. (**a**) Chemical structure of rabeprazole before and after activation at acidic pH^37^. R indicates the position of the disulfide bonded protein. (**b**) HTS assay in the presence of rabeprazole that was either not pre-activated (grey bars) or pre-activated in acidic buffer (dashed bars). (**c**) Inhibition of the LEM-BAF-DNA chain by rabeprazole (100 µM) under oxidizing (w/o GSH) or reducing (w/ GSH) conditions in the HTS assay. Rabeprazole sulfide was used as a control since it cannot form disulfide bridges. (**d**) The effect of acid activated rabeprazole on the DFR assay, which was performed with the DNA restriction enzyme HindIII. (**e**) ESI–MS spectra for the BAF protein after incubation with rabeprazole under reducing (w/ GSH) and oxidizing (w/o GSH) conditions. The theoretical masses of the compound-BAF adducts are indicated in the inset. (**f, g**) Fluorescence-activated cell sorting (FACS) analysis of HeLa cells 24 h after transfection with a GFP encoding plasmid using the transfection agent X-tremeGENE 9. The transfection was performed in the presence of rabeprazole (100 µM, with or without acid activation), using rabeprazole-free and uncomplexed DNA transfections as positive and negative controls, respectively. Results are shown as the percentage of GFP positive cells (f) and the GFP intensity normalized to the positive control (g). Statistical significance was tested with respect to the positive control. NS = not significant. B-D: Fluorescence was normalized with respect to the fluorescence intensity in absence of rabeprazole. B, C, D F, G: Mean ± SD (N = 3).

One of the compounds in the group of true positive hits was the proton pump inhibitor rabeprazole. Upon acid activation, rabeprazole undergoes a structural rearrangement and then forms a disulfide bridge with exposed cysteines of the gastric H^+^,K^+^-ATPase (Fig. 3a)^37,38^. In fact, the inhibitory activity of rabeprazole in the HTS assay was strongly increased after preincubation in an acidic buffer (Fig. 3b). Therefore, we hypothesized that disulfide bridge formation might be responsible for the inactivation of the BAF protein, considering that BAF contains four cysteine residues in its C-terminal alpha helix whereas the LEM-domain protein (His_6_-MBP-LEM) does not contain any cysteine residues. To evaluate this hypothesis, we manually performed the HTS assay under oxidizing conditions (without glutathione (GSH)), reducing conditions (with GSH) and with the rabeprazole metabolite rabeprazole sulfide that cannot undergo acid activation and disulfide bridge formation^39^. Clearly, the inhibitory activity of rabeprazole was lost in the presence of GSH as well as when rabeprazole sulfide was used, indicating that disulfide bridge formation with BAF is relevant for the inhibition of DNA retention (Fig. 3c).

We then tested rabeprazole with the DFR assay to investigate whether it was interfering with the BAF-DNA or rather the LEM-BAF interaction. Since the restriction enzyme EcoRI contains a cysteine residue, the assay was performed with the cysteine-free restriction enzyme HindIII. The latter was shown to retain its DNA hydrolyzation activity in the presence of rabeprazole (Fig. 3d: BAF-/HindIII+/Rabeprazole+). Interestingly, rabeprazole did not result in a signal reduction of the DFR assay (Fig. 3d: BAF+/HindIII+/Rabeprazole+), indicating that rabeprazole adduct formation does not interfere with the DNA binding site of BAF but rather affects its interaction with the LEM-domain.

Furthermore, we performed ESI–MS analysis of the BAF rabeprazole mixture both under oxidizing and reducing conditions (Fig. 3e). Clearly, the acid activated conformation of rabeprazole forms stable protein adducts only in the absence of GSH, indicating that this interaction is due to disulfide bridge formation. The measured masses for the BAF monomer with up to 4 rabeprazole adducts were 10,395.5, 10,738, 11,081 and 11,422 Da, which are close to the theoretical masses being 10,402, 10,745.5, 11,088.9 and 11,432.3 Da, respectively. Therefore, one BAF monomer was bound by up to four rabeprazole molecules, which is the number of cysteines present in BAF.

As a last step in the evaluation of rabeprazole, we tested whether it enhances the DNA transfection efficiency of HeLa cells (Fig. 3f,g). The compound was applied both in the activated and non-activated conformation to the cells, which were subsequently transfected with an enhanced green fluorescent protein encoding plasmid (pEGFP), complexed with X-tremeGENE 9 transfection agent. In both cases, however, no positive effect on transfection efficiency was observed.

#### True positive hits of Maybridge library

**Figure 4:**
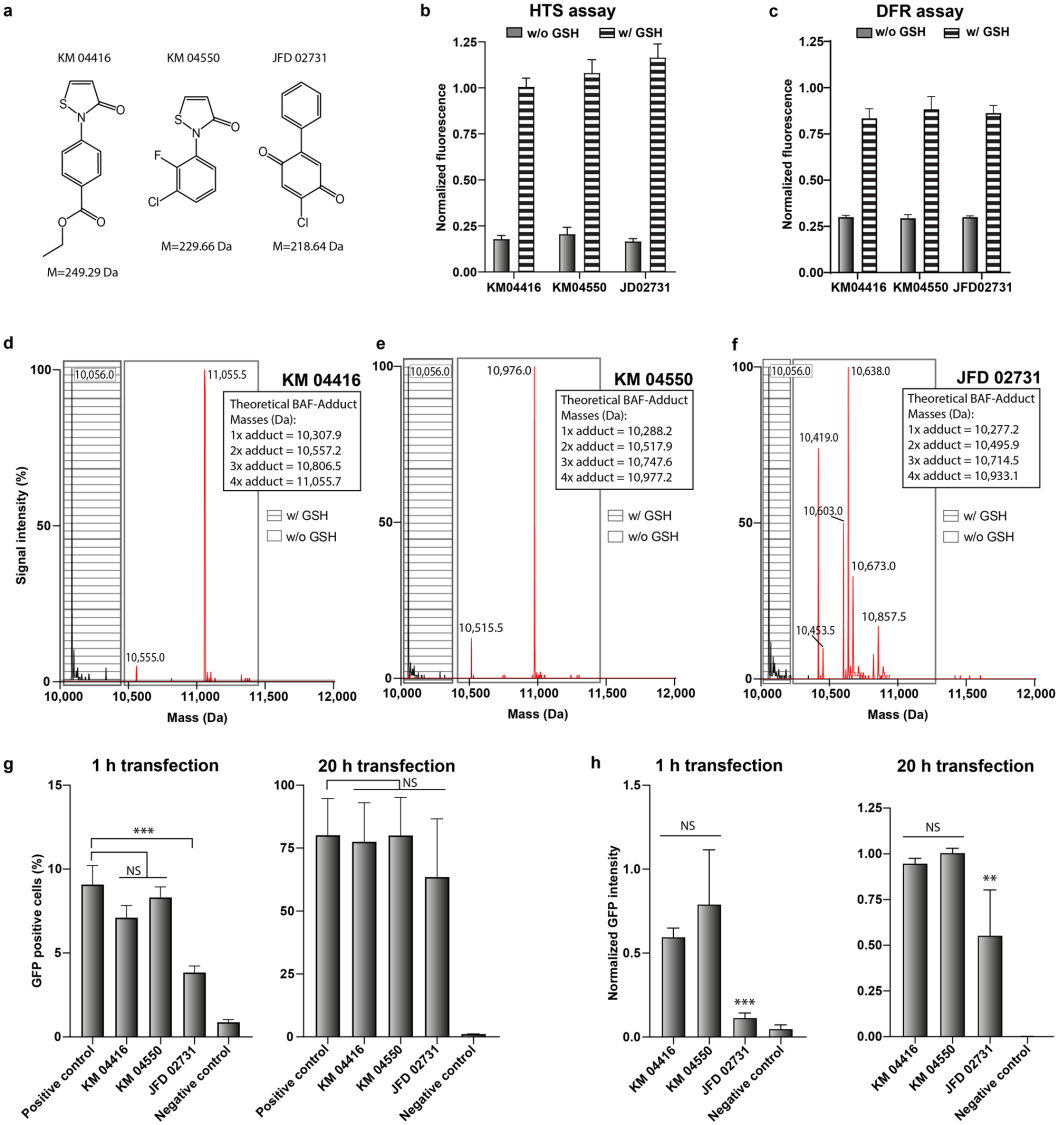
Validation of the Maybridge hits as BAF inhibitors. (**a**) The molecular structures and masses of the three Maybridge library hits: KM 04416, KM 04550 and JFD 02731. (**b**,**c**) Inhibitory effect of the Maybridge hits in the HTS (b) and DFR (c) assays (concentration 10 µM) under oxidizing (w/o GSH) and reducing (w/ GSH) conditions. The fluorescence was normalized with respect to the positive control (DMSO) under oxidizing and reducing conditions, respectively. (**d**–**f**) ESI–MS analysis of BAF adduct formation under reducing (w/ GSH, black, dashed box) and oxidizing (w/o GSH, red, clear box) conditions with the Maybridge hits KM 04416, KM 04550 and JFD 02731, respectively. (**g**,**h**) FACS analysis of HeLa cells 24 h after the transfection with a GFP encoding plasmid for 1 h or 20 h using the transfection agent X-tremeGENE 9. The transfection was performed in the presence of KM 04416 (10 µM), KM 04450 (10 µM) or JFD 02731 (50 µM). The subfigures depict the percentage of GFP positive cells and the mean GFP intensities, respectively. B, C, G, H: Mean ± SD (N = 3). *p < 0.05, **p < 0.01, ***p < 0.01, NS = not significant respective to positive control.

The structure of the three true positive hits of the Maybridge library (KM 04416, KM 04450 and JFD 02731) are shown in Fig. 4a. These compounds were further evaluated, similarly to the procedure followed for rabeprazole. The manual repetition of the HTS assay indicated that these compounds were also active only under oxidizing conditions, and lost their activity in the presence of physiological GSH concentrations (Fig. 4b). Unlike rabeprazole, all three Maybridge hits inhibited DNA fragment retention in the DFR assay under oxidizing conditions (Fig. 4c).

The ESI–MS analysis revealed the formation of stable BAF adducts under oxidizing conditions when a compound-to-BAF molar ratio of 10:1 was applied (Fig. 4d–f). For KM 04416, we observed a dominant adduct peak of 11,055.5 Da, corresponding to the theoretical mass of the BAF monomer with four adducts (11,055.7 Da). A minor peak with 10,555.0 Da matches the theoretical mass of BAF with two adducts (10,557.2 Da), while the wild type BAF species (10,056 Da) disappeared completely (Fig. 4d). Similarly, KM 04550 formed four adducts per BAF monomer, as indicated by the 10,976.0 Da peak that matches the theoretical mass of 10,977.2 Da (Fig. 4e). As in the case of KM 04416, a minor peak corresponding to BAF with two adducts was detected at 10,515.5 Da (Fig. 4e).

Interestingly, JFD 02731 resulted in BAF adduct formation, too (Fig. 4f). This was not expected, since JFD 02731 does not contain any thiol groups and therefore would not be able to form disulfide bridges. However, adduct formation could still take place with electrophilic centers in the molecule that can be attacked by nucleophilic cysteine residues (e.g., Michael addition)^40^. The resulting masses can only be explained by the loss of chloride ions in the ionization process. More specifically, the first two adduct peaks (10,419.0 and 10,453.5 Da) could represent a BAF monomer with two JFD 02731 adducts (theoretical mass 10,495.9 Da), of which one (10,460.5 Da) or both (10,425 Da) molecules were dechlorinated, respectively. These are followed by three peaks (10,603.0, 10,638.0 and 10,673 Da) representing BAF with three adducts (theoretical mass 10,714.5 Da) after dechlorination of 1, 2 or all the 3 attached compounds. The heaviest peak with 10,857.5 Da could accordingly comprise a BAF species with four JFD 02731 adducts (10,933.1 Da), of which 2 have been dechlorinated. For all compounds, adduct formation was prevented in the presence of GSH (dashed boxes in Fig. 4d–f).

Finally, we tested if the three compounds could enhance the transfection efficiency of HeLa cells (Fig. 4g,h). The cells were transfected for 1 and 20 h with pEGFP in presence of the compounds. FACS analysis 24 h after transfection indicated that none of the compounds significantly increased the efficiency of transgene expression nor the percentage of GFP expressing cells. In fact, JFD 02731 even led to a significant decrease in the transfection efficiency in terms of both the percentage of GFP expressing cells and the GFP signal intensity. This effect was more pronounced after 1 h than after 20 h transfection experiments.

## Discussion

BAF is a fascinating little protein that contributes in many ways to the survival of mammalian cells. It is not only involved in the reformation of the nucleus after cell division, but also in the organization of chromatin, the mitotic spindle, viral DNA protection and cytoplasmic DNA clustering. Inhibitors against the different BAF functions could therefore be of great interest, not only for DNA transfection applications, but also as anti-cancer therapeutics as well as anti- or pro-viral agents.

In this manuscript, we described a new protocol to purify the wild type BAF protein in reasonable quantities from *E. coli* bacteria and developed two robust ELISA-type assays for the screening of inhibitors of BAF-dependent DNA retention. The HTS assay involves the LEM-BAF-DNA interaction chain, and therefore allows to find effectors of cytoplasmic membrane cage formation. The DFR assay depends only on the BAF-DNA interaction and is thus used to distinguish between LEM-BAF and BAF-DNA interaction inhibitors. We expect that an inhibitor against the DNA binding function of BAF would be highly cytotoxic, due to the many functions of BAF in nuclear envelope assembly, genome organization, stability and DNA repair. Paquet et al*.*, for example, demonstrated that BAF with an alanine to threonine mutation in residue 12 has a lower DNA binding affinity, which causes nuclear defects and is the genetic basis for the Néstor-Guillermo progeria syndrome (NGPS)^41^. The LEM-BAF interaction, on the other hand, is more redundant, since there are several transmembrane proteins that are able to bind DNA directly or via different linker proteins, e.g., histones^42^. Such an inhibitor would thus be expected to have lower cytotoxicity and be more suitable as an enhancer for gene delivery formulations.

We demonstrated that the HTS assay is robust and can be used in an automatized setting. By screening 6 small compound libraries, we found that BAF is susceptible to form disulfide bonds resulting in the inactivation of its DNA clustering activity. While this might give rise to new insights on the potential regulation of this protein, there was no positive effect on DNA transfection efficiency observed after inhibitor treatment of HeLa cells. This is probably due to the reducing nature of the cytoplasmic environment, in which the disulfide bond-based adduct formation between BAF and the identified inhibitors cannot occur efficiently. Furthermore, the high abundance of cysteine residues on the cell surface would probably lead to an unspecific action of those molecules. The latter might also explain the negative impact on transfection efficiency in the presence of JFD 02731.

Interestingly, the mechanism of the HTS assay inhibition seems to differ among the analyzed compounds. While rabeprazole, KM 04416, KM 04550 and JFD 02731 are clearly forming adducts with BAF, only the latter three also inhibit the DNA fragment retention in the DFR assay. Rabeprazole, on the other hand, seems to block the interaction of BAF with the LEM-domain protein. However, it remains unclear if any of the identified inhibitors interfere with BAF in a structure-dependent way, or just by sterical interference after adduct formation. JFD 02731 must be categorized as a pan-assay interference compound (PAIN)^43^, due to its quinone-like functional group that is involved in non-specific adduct formation with electrophilic residues^44^. Also the isothiazolone groups in KM 04416 and KM 04550 were already described to be reactive with cysteines^45^. Rabeprazole itself has, to our knowledge, not been categorized as a PAIN. Due to its capacity to form disulfide bridges after acid activation, it should, however, be treated carefully in protein assays with oxidizing conditions.

## Conclusion

We successfully developed two assays for the monitoring of the DNA clustering function of BAF and demonstrated in a moderately sized screening campaign that these can be used to detect effectors of the LEM-BAF-DNA interaction chain. In order to find more suitable inhibitors, larger compound libraries—potentially with higher molecular weight compounds—should be screened. Additionally, we showed that the assays can be applied to study enzymatic effectors of BAF and its interaction partners, which will allow to better understand the cellular roles of this multifunctional system in the context of cytoplasmic DNA retention and beyond.

## Methods

### Materials

A detailed materials list is provided in the Supplementary Methods.

### DNA construct cloning

#### Vector 1

The plasmid pET His6 TEV LIC (1B) was modified by insertion of a multiple cloning site, a hexahistidine (His_6_) tag, a TEV protease cleavage site and the sequence of wild type BAF (Uniprot: BAF_HUMAN) (Supplementary Fig. S9). The insert was generated by GeneArt Gene Synthesis (Thermo Fisher Scientific, Waltham, MA) and inserted into the plasmid at the restriction sites XbaI and XhoI. This plasmid containing the wild type BAF sequence is referred to as vector 1 and was used to clone the constructs (1) His_6_-MBP-BAF VRK1, (2) His_6_-MBP-BAF G27E VRK1, (3) MBP-Lambda phosphatase and (4) MBP-LEM as described in the Supplementary Methods.

### Recombinant protein expression

#### BAF and BAF G27E expression and purification

His_6_-MBP-BAF or His_6_-MBP-BAF G27E were coexpressed with the kinase VRK1 (uniprot: VRK1_HUMAN, His_6_-MBP-BAF VRK1 construct) in *E. coli* BL21(DE3)pLysS in the inactive hyperphosphorylated state, with the maltose binding protein (MBP, uniprot: MALE_ECOLI) as a solubility tag. The bacteria were grown at 37 °C to an OD_600_ of 0.5, induced with isopropyl β-D-1-thiogalactopyranoside (IPTG, 0.4 mM), followed by shaking at 37 °C for 3 h. Then the cells were pelleted at 4 °C, 4000×*g* for 10 min. The cell pellet was frozen at − 20 °C. The next day the pellet was thawed on ice for 30 min and resuspended in lysis buffer (HEPES (20 mM, pH 7.4), KCl (150 mM), glycerol (10%), MgCl_2_ (10 mM)) that was supplemented with Protease Inhibitor Cocktail and lysozyme (1 mg/mL). After incubation at room temperature (RT) for 30 min, the cells were lysed by sonication on ice. The lysate was cleared by centrifugation at 20,000×*g*, 4 °C for 45 min. The supernatant was collected, filtered through a 0.22-µm pore size filter and loaded on a nickel-nitrilotriacetic acid (Ni–NTA) column that was equilibrated with lysis buffer supplemented with imidazole (10 mM). After thorough washing of the column, the protein was eluted in lysis buffer supplemented with imidazole (250 mM).

A fresh batch of lambda phosphatase was prepared in parallel with the same protocol but with a modified lysis buffer (HEPES (50 mM, pH 7.5), NaCl (0.2 M), imidazole (5 mM), glycerol (10%), MnCl_2_ (1 mM)). Then, the phosphatase and BAF were mixed in a weight ratio of 1:1 in dephosphorylation/TEV cleavage buffer (HEPES (20 mM, pH 7.5), NaCl (1 M), glycerol (10%), MnCl_2_ (1 mM), ditihothreitol (DTT, 2 mM), and TEV protease (1:100, w/w)). After overnight incubation at 30 °C, a buffer exchange into lysis buffer was performed to remove the imidazole from the sample. The protein sample was again supplemented with imidazole (20 mM) and loaded on a Ni–NTA column, whereas this time the column flow-through was collected containing the dephosphorylated and tag-less wild type BAF. The sample was concentrated and loaded on a Superdex 75 10/300 GL column (GE Healthcare, Chicago, IL) with a flow rate of 0.4 mL/min in running buffer (HEPES (20 mM, pH 7.4), KCl (150 mM), glycerol (10%)). The fractions containing wild type BAF were pooled, aliquoted, snap frozen in liquid nitrogen and stored at − 80 °C. The protein concentration was estimated by spectrophotometry at 280 nm (NanoPhotometer Pearl, Implen GmbH, Munich, Germany) with an extinction coefficient of 14,230 M^−1^ cm^−1^ and a theoretical molecular mass of 10,059 Da. Physiochemical parameters of all proteins were calculated using the ProtParam (ExPASy, Bioinformatics Resource Portal, Switzerland) online tool.

#### MBP-LEM expression and purification

The MBP-LEM protein was expressed in *E. coli* BL21(DE3)pLysS according to the same protocol as for the lambda phosphatase, but without MnCl_2_ supplementation of the purification buffers. The eluate from the Ni–NTA column was stored at − 80 °C without removal of the His_6_-MBP solubility tag. Protein concentration was estimated by spectrophotometry at 280 nm using and extinction coefficient of 73,800 M^−1^ cm^−1^ and a theoretical molecular mass of 52,266 Da (parameters calculated with ProtParam).

### Assay protocols

#### ATTO 425-labelled and biotinylated DNA

The linear 270-bp DNA molecules were generated by PCR from a non-coding DNA sequence. The PCR template (Supplementary Fig. S4) was produced by GeneArt Gene Synthesis and inserted into vector 1 using the restriction enzymes XbaI and XhoI. From this plasmid, a 405-bp DNA sequence was amplified using the primers 5′-GGGGAATTGTGAGCGGATA-3′ and 5′-TTCGGGCTTTGTTAGCAG-3′ by Phusion High-Fidelity DNA Polymerase (New England Biolabs). The PCR product was purified by QIAquick PCR Purification Kit (Qiagen) according to the manufacturer protocol, and used as a template for a nested PCR (GoTaq Green Master Mix, Promega) with the primers 5′-CACCATCACCATGAAAACCT-3′ (forward) and 5′-TCGAGAGGTCAGGGTGGTCA-3′ (reverse) to generate a 270 bp PCR product. For the HTS assay, both nested primers were modified with a 5′ ATTO 425 dye, resulting in a PCR product with fluorescent labels on both termini. For the DFR assay, only the nested reverse primer carried the ATTO 425 modification and the nested forward primer was 5′ biotinylated.

The nested (biotinylated and/or fluorescently labelled) PCR products were purified by ethanol precipitation as follows: the PCR product was mixed with ammonium acetate (7.5 M) solution in a ratio of 1:6 (V_NH4Ac_/V_PCR_). Then, EtOH was added in a ratio of 2.5:1 (V_EtOH_/V_sample_). After centrifugation for 10 min at 18,000×*g* and 4 °C, the supernatant was discarded, the DNA pellet was washed with wash buffer (potassium acetate (10 mM, pH 5.0), EtOH (80%), EDTA (16.7 µM)) and centrifuged as before. The washing step was performed three times. Then, the DNA pellet was dried at 50 °C and resuspended in resuspension buffer (Tris–HCL (10 mM, pH 7.5), EDTA (1 mM)). The DNA concentration was determined by spectrophotometry (NanoPhotometer Pearl, Implen GmbH).

### HTS assay

The HTS assay was automatized and the screening performed at the Biomedical Screening Facility (BSF) at École Polytéchnique Fédérale Lausanne (EPFL). MBP-LEM (10 pmol) protein in phosphate buffered saline (PBS, 30 µL), were added to each well of a black 386-well High Bind Plate (Corning, NY) using an EL406 Washer Dispenser (BioTek, Winooski, VT). The plate was covered with a plastic foil and incubated over night at 4 °C. The wells were then washed 3 times with PBS (90 µL) using the EL406 Washer, and subsequently blocked with PBS (90 µL, supplemented with BSA (1%)). After incubation for 60 min, the plate was washed 3 times with PBST (90 µL, PBS supplemented with polysorbate 20 (0.05%)). Then, BAF (5 pmol) in PBST (30 µL) were added to each well, followed by the compound in the appropriate concentration. The compound libraries were previously transferred from Echo microplates into REMP microplates using an acoustic liquid handler (Echo 550 Liquid Handler, Labcyte Inc.) and were then added to the assay plate with the Sciclone ALH 3000 Advanced Liquid Handler (Caliper Life Sciences, Hopkinton, MA) using 25 µL Sciclone tips.

In order to perform the assay under reducing conditions, reduced GSH (2 mM final concentration) was added to the BAF sample (30 µL). The plate was incubated for 60 min at RT. Then, ATTO-labelled DNA (0.75 pmol) in PBST (10 µL) was added. After 60 min of incubation at RT in the dark, the plate was again washed 3 times with PBS. 3—4 h after the last washing step the fluorescence intensity (excitation: 436 nm, emission: 484 nm) was measured in a final volume of PBS (50 µL) using a Synergy NEO HTS Microplate Reader (BioTek).

The same protocols as above were applied when the HTS assay was performed manually with the only difference that the pipetting/washing steps were performed with a 125 µL VOYAGER multichannel pipet (Integra Biosciences, Zizers, Switzerland), and the readout was conducted on a TECAN Infinite M200 plate reader (Tecan AG, Mannedorf, Switzerland), with an excitation and emission bandwidth of 9 and 20 nm, respectively.

The signal strength of each assay plate was quantified by determining the Z’-factor according to^36^. The first 2 columns (32 wells) of each plate were filled with positive controls (DMSO added instead of compound) and the last 2 columns with negative controls (PBST added instead of BAF). The standard deviation (σ) of the positive controls was then used to determine the threshold for considering a compound as a “hit”. More specifically, when the signal intensity (mean of duplicate values) of a compound deviated more than ± 3σ from the mean signal intensity of the positive controls on the same plate, then this compound was considered as a hit. The compounds were tested in duplicates on separate plates.

#### DNA fragment retention (DFR) assay

A Pierce streptavidin coated black 96-well plate (Thermo Fisher Scientific) was washed 3 times with wash buffer (HEPES (20 mM, pH 7.4), NaCl (150 mM), BSA (0.1%), polysorbate 20 (0.05%)). Then, biotinylated and ATTO 425-labelled DNA (2 pmol) in wash buffer (100 µL) were added to each well. After incubation for 3 h at RT the plate was again washed 3 times as above. The restriction enzyme EcoRI FD (1 µL), BAF (10 pmol) and the compound of interest were then premixed in a total volume of 50 µL restriction enzyme buffer (Tris (50 mM, pH 7.5), MgCl_2_ (10 mM), NaCl (100 mM), BSA (0.1%)) and added to the well after removing the wash buffer. The plate was incubated for 1 h at 37 °C, followed by 2 washing steps with analysis buffer (HEPES (20 mM, pH 7.4), NaCl (150 mM)). ATTO 425 fluorescence was then measured as above on a TECAN Infinite 200 plate reader in a final volume of 100 µL per well.

The quenching effect of fluorescent DNA intercalators was measured with the DFR assay. The biotinylated and ATTO 425-labelled DNA was immobilized on a plate as above. Then 10 µM DNA intercalator were added per well, followed by 30 min incubation at room temperature. Finally, the wells were washed 3 times with PBS and ATTO 425 fluorescence was measured as described above.

### Cell culture and transfection

HeLa cells (passage number 3–30, tested negative for mycoplasma contamination, MycoAlert Kit, Lonza AG, Basel, Switzerland) in complete growth medium (DMEM GlutaMAX, FBS (10%), Pen/strep (1%)) were seeded on 24-well plates and grown to a confluence of about 60% on the day of transfection. The transfection was performed using X-tremeGENE 9 transfection reagent, according to the manufacturer instructions. 30 min prior to transfection, fresh complete medium supplemented with the compound of interest was added to the cells. Then, complexed plasmid DNA (100 ng) in Opti-MEM (Opti-MEM I Reduced-Serum Medium, Thermo Fisher Scientific, 10 µL) were applied per well, followed by 1 or 20 h incubation in typical cell culture conditions (37 °C, CO_2_ (5%), humidified atmosphere). Then, the cells were washed 3 times with PBS, and fresh complete medium, supplemented with the compound of interest, was added for further incubation. Between 20 and 24 h after transfection, the cells were processed for FACS analysis.

### Fluorescence-activated cell sorting (FACS)

HeLa cells growing on a 24-well plate were washed 4 times with PBS, and subsequently trypsinized for 2 min at 37 °C. The cells were collected in 4 °C complete growth medium and pelleted by centrifugation at 300×*g* for 10 min at 4 °C. The pellet was resuspended in ice cold FACS buffer (PBS, EDTA (2 mM), BSA (0.05%), 150 µL) and transferred to a Costar round-bottom 96-well assay plate (Corning) for FACS analysis (CytoFLEX Flow Cytometer, Beckman Coulter Life Sciences, Nyon, Switzerland). For each measurement 10,000 cells were collected and analyzed using the FlowJo software (Tree Star Inc., Ashland, OR).

### Electrospray ionization–mass spectrometry (ESI–MS)

BAF phosphorylation was conducted by incubation of BAF (5 µM) with VRK1 (0.1 µM) in 1 × T4 DNA ligase buffer (New England Biolabs) for 30 min at 37 °C. Subsequently, the samples were stored on ice until analysis. BAF adduct formation was studied by mixing BAF (5 µM) with rabeprazole (10 µM) or the Maybridge compounds (50 µM) in PBS buffer. To achieve reducing conditions the buffer was supplemented with reduced GSH (2 mM). Rabeprazole was preincubated for 30 min at RT in activation buffer (acetate (20 mM, pH 4.0), NaCl (150 mM)). The MS samples were incubated at RT for 30 min, then stored on ice until analysis.

Prior to ESI–MS analysis, samples were desalted using C4 ZipTips (Merck Millipore, Burlington, MA) and analyzed in methanol:2-propanol:0.2% formic acid (30:20:50 v/v). The solution was infused at a flow rate of 1 µL/min and sprayed using Fused-silica PicoTip emitters (New Objective, Woburn, MA) with capillary ID of 75 μm and tip ID of 30 μm. Nano ESI–MS analyses of the samples were performed on a SYNAPT G2-Si mass spectrometer and the data were recorded with the MassLynx 4.2 Software (both Waters, UK). Mass spectra were acquired in the positive-ion mode by scanning an m/z range from 100 to 5000 Da with a scan duration of 1 s and an interscan delay of 0.1 s. A spray voltage (3 kV), a cone voltage (40 V) and a source temperature (80 °C) were used. The recorded m/z data were deconvoluted into mass spectra by applying the maximum entropy algorithm MaxEnt1 (MaxLynx) with a resolution of 0.5 Da/channel for the output mass and a Uniform Gaussian Damage Model at the half height of 0.7 Da.

### Circular dichroism

Circular dichroism (CD) spectra were measured using a Jasco J-710 CD spectrometer (JASCO Deutschland GmbH, Pfungstadt, Germany). The proteins BAF (0.15 mg/mL) and MBP-LEM (0.4 mg/mL) were measured 5 times in PBS from 260–200 nm, in the continuous scanning mode at a scanning speed of 50 nm/min, 1 s response time and a 2 nm bandwidth. A PBS blank was recorded and subtracted from the protein spectra. The resulting curve was smoothened by averaging over 5 neighboring points with a second order smoothing polynomial function using the GraphPad Prims software version 8 (GraphPad Softwares, La Jolla, CA) (Supplementary Fig. S10).

### Statistics

Statistical analysis was performed using the GraphPad Prims software version 8. All data are generally represented as mean with standard deviation (SD) of at least 3 independent experiments. Significance was tested by one-way ANOVA combined with the Tukey’s multiple comparisons method.

## Supplementary information

Supplementary Information.

## Data Availability

The datasets generated during and/or analysed during the current study are available from the corresponding author on reasonable request.
