# Supplementary material for: DNA unchained: two assays to discover and study inhibitors of the DNA clustering function of barrier-to-autointegration factor
Source: Sci Rep. 2020 Jul 23;10:12301. doi: 10.1038/s41598-020-69246-x (PMC7378220; doi:10.1038/s41598-020-69246-x)
Supplement: Supplementary file 1 — Supplementary Information. [file 41598_2020_69246_MOESM1_ESM.docx]

**Supplementary Information**

**DNA unchained: Two assays to discover and study inhibitors of the DNA clustering function of barrier-to-autointegration factor**

Michael Burger^1^, Caroline Schmitt-Koopmann^1^, Jean-Christophe Leroux^1*^ (jleroux@ethz.ch)

^1^Institute of Pharmaceutical Sciences, ETH Zurich, Zurich, Switzerland

**Supplementary Methods**

## Materials

pET His6 TEV LIC cloning vector (1B) was a gift from Scott Gradia (Berkley, CA, Addgene plasmid #29653). pRK793 was a gift from David Waugh (Frederick, MD, Addgene plasmid #8827). pPET-PKR/PPase was a gift from James Cole (Storrs, CT, Addgene plasmid #42934). pDONR223-VRK1 was a gift from William Hahn & David Root (Boston, MA, Addgene plasmid #23496). The compounds KM 04416 (IUPAC: ethyl 4-(3-oxo-1,2-thiazol-2(3H)-yl)benzoate), KM 04550 (IUPAC: 2-(3-chloro-2-fluorophenyl)-1,2-thiazol-3(2H)-one) and JFD 02731 (IUPAC: 2-chloro-5-phenyl-1,4-benzoquinone), as well as phosphate buffered saline (PBS, pH 7.4), FastDigest restriction enzymes and synthesized double stranded DNA sequences (GeneArt Gene Synthesis service) were purchased from Thermo Fisher Scientific (Waltham, MA). The Calf Intestinal Phosphatase (CIP) and Phusion High-Fidelity DNA Polymerase was obtained from New England Biolabs (Ipswitch, MA). DNA oligonucleotides were synthesized by Microsynth AG (Balgach, Switzerland). Chemically competent *E. coli* DH5alpha and BL21(DE3)pLysS cells and GoTaq G2 HotStart Green Master Mix were purchased from Promega AG (Dübendorf, Switzerland). HeLa (ATCC CCL-2) cells were obtained from ATCC (Manassas, VA). Rabeprazole and rabeprazole sulfide were purchased from Tokyo Chemical Industry (Montgomeryville, PA). Protease Inhibitor Cocktail and X-tremeGENE 9 transfection agent were obtained from Sigma Aldrich Chemie GmbH (Buchs, Switzerland) and nickle-nitrilotriacetic acid (Ni-NTA) agarose from Qiagen (Germantown, MD). pEGFP plasmid (pc3DNA) was purchased from Addgene (Cambridge, MA).

For the robotic platform: Echo Qualified 384-Well Polypropylene Microplate, Clear, Flat Bottom, P-05525 were purchased from Labcytes Inc. (Sunnyvale, CA), Sciclone tips (25 μL, 3^rd^ Gen) were obtained from Caliper Life Sciences (Hopkinton, MA) and REMP Storage Microplates from Brooks life sciences (Chelmsford, MA). Easyseal Plate Sealer plastic foils were purchased from Greiner Bio-One (Frickenhausen, Germany). The following chemical libraries were screened: the Pfizer Licensed Compound Library (Pfitzer, New-York, NY), the Prestwick Chemical Library (Prestwick Chemicals, Illkirch, France), the Protein–Protein Interaction Library (Life Chemicals, Niagara-on-the-Lake, Canada); the Chemical Diverse Collection (BSF selection made from Enamine (Riga, Latvia), Life Chemicals and Chemdiv (San Diego, CA)), the Natural Products Library (InterBioscreen, Moscow, Russia) and the Maybridge Library (Maybridge Chemical Company, Altrincham, UK); All other chemicals were obtained from Sigma Aldrich Chemie.

## DNA construct cloning

## Vector 1

The plasmid pET His6 TEV LIC (1B) was modified by insertion of a multiple cloning site, a hexahistidine (His_6_) tag, a TEV protease cleavage site and the sequence of wild type BAF (Uniprot: BAF_HUMAN) (**Supplementary** **Fig. S9**). The insert was generated by GeneArt Gene Synthesis and inserted into the plasmid at the restriction sites XbaI and XhoI. This plasmid is referred to as vector 1.

## 2.2 His_6_-MBP-BAF VRK1 and His_6_-MBP-BAF G27E VRK1 constructs

Vector 1 was linearized with HindIII FD and XhoI FD, and subsequently dephosphorylated with the CIP phosphatase. The maltose binding protein (MBP, uniprot: MALE_ECOLI) gene with a N-terminal hexahistidine tag was amplified by polymerase chain reaction (PCR, Bio Rad PTC-200 Thermal Cycler, Hercules, CA) from the template plasmid pRK793 using the primers 5’-AAGGGAAGCTTCGATGAAGATCGAAGAAGGTAAACTGG-3’ and 5’-GCGGGATCCGATCCCGAGGTTGTTGTTATT-3’ and digested with HindIII FD and BamHI FD. Further, a DNA insert encoding human VRK1 (uniprot: VRK1_HUMAN) was inserted downstream of the BAF construct with an internal ribosomal binding site as follows: a spacer containing a ribosomal entry site was generated by PCR from the template pPET-PKR/PPase using the primers 5’-ACACATGTTAGAGCCCTTC-3’ and 5’-CCGCTCGAGGCTGCCATCAATTTTTTCG-3’. The spacer insert was digested with EcoRI FD and NdeI FD. The VRK1 insert was generated by PCR from the template plasmid pDONR223-VRK1 using the primers 5’-GGGAATTCCATATGCCTCGTGTAAAAGCA-3’ and 5’-CCGCTCGAGTTACTTCTGGACTCTCTTTCT-3’, and then digested with NdeI FD and XhoI FD. The 4 inserts (MBP, BAF, spacer, VRK1) were ligated into the linearized vector 1 and transformed into chemically competent *E. coli* DH5α. A single clone was expanded and the resulting plasmid minipreparation was sequenced over the full insert region. Once the sequence was verified, the plasmid was retransformed into chemically competent *E. coli* BL21(DE3)pLysS for protein expression.

The BAF G27E mutant construct was generated according to the same protocol, starting with the mutated BAF DNA sequence (generated by GeneArt Gene Synthesis, **Supplementary Fig. S9**).

## MBP-Lambda phosphatase plasmid

Vector 1 was linearized using the restriction enzymes HindIII FD and EcoRI FD, and subsequently dephosphorylated using the CIP phosphatase. The lambda phosphatase gene was amplified by PCR from the plasmid pPET-PKR/PPase using the following primers: 5’-CGCGGATCCGGACAGATGCGCTATTACG-3’ and 5’-CCGGAATTCTTCCTTTCGGGCTTTGTTA-3’. The PCR product was digested with the restriction enzymes BamHI FD and EcoRI FD. The phosphatase gene was inserted downstream of a His_6_-MBP tag (analog to the MBP-BAF yPP construct). The plasmid was amplified in DH5a, sequenced and retransformed into *E. coli* BL21(DE3)pLysS, as described above.

## MBP-LEM plasmid

Vector 1 was linearized with the restriction enzymes HindIII FD and EcoRI FD, and dephosphorylated by CIP phosphatase. The MBP gene insert with an N-terminal hexahistidine tag and TEV cleavage sequence was inserted as in the MBP constructs above. The LEM sequence from human emerin (coding for amino acids 1-47, uniport: EMD_HUMAN, **Supplementary Fig. S11**) was obtained by GeneArt Gene Synthesis, digested with BamHI FD and EcoRI FD, and ligated as described above.

**Supplementary Figures**


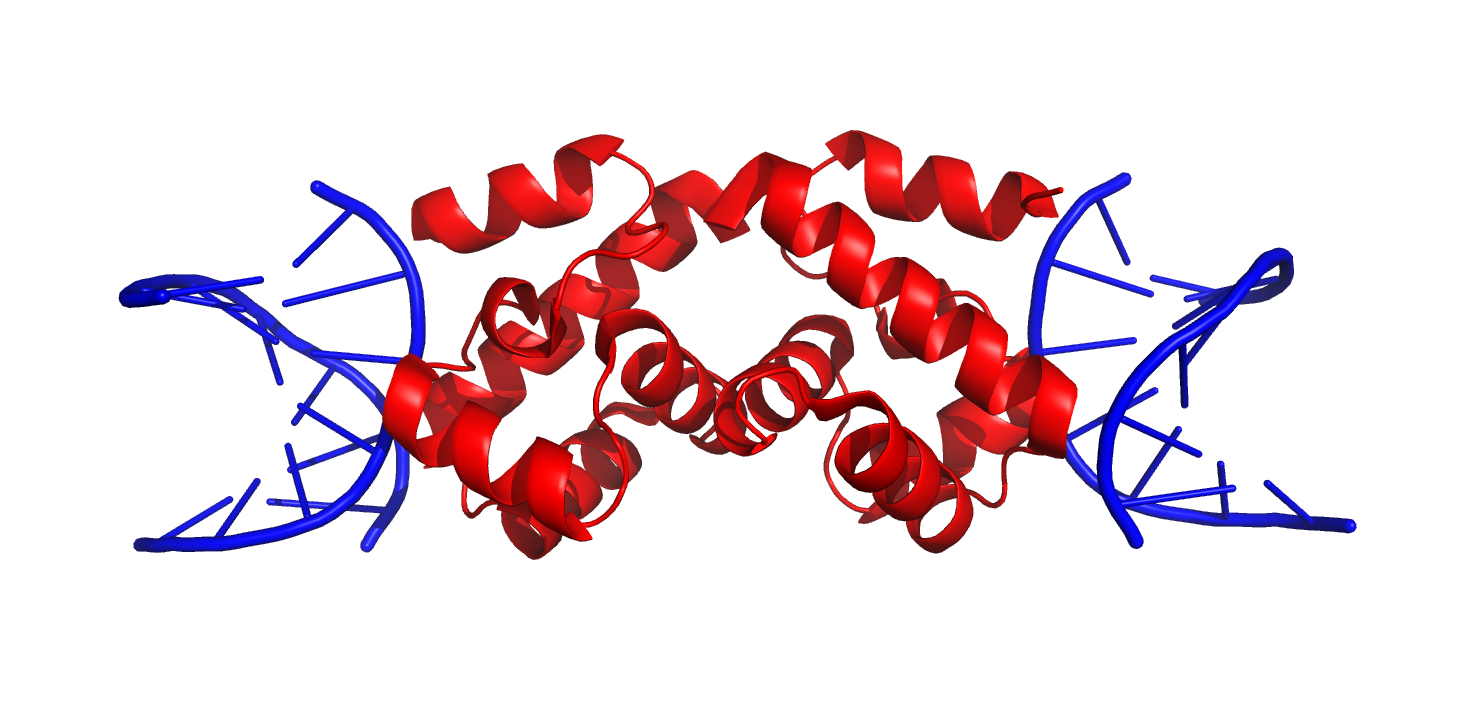


**Figure S1.** Crystal structure of the BAF homodimer (red) bound to two DNA strands (blue). Figure generated with Pymol (Schrödinger, NY) from PDB structure 2bzf^1^.

**
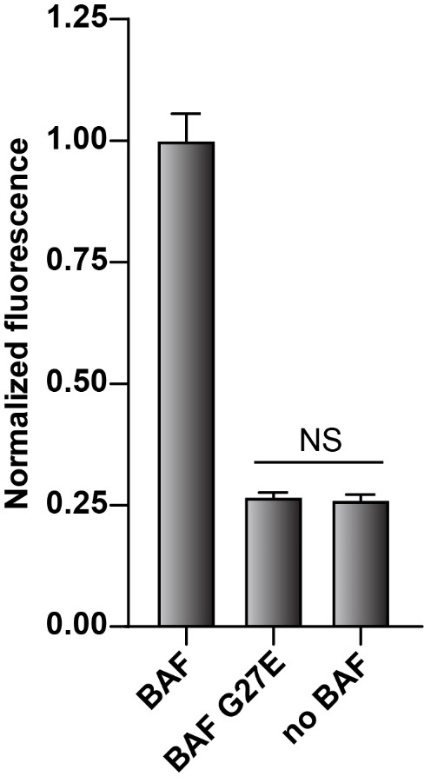
**

**Figure S2.** The HTS assay was performed with BAF (200 nM), the non-DNA binding BAF mutant G27E or without BAF. No significant (NS) difference in fluorescent DNA retention was observed when applying either the BAF mutant or no BAF. Mean + SD (N=3)


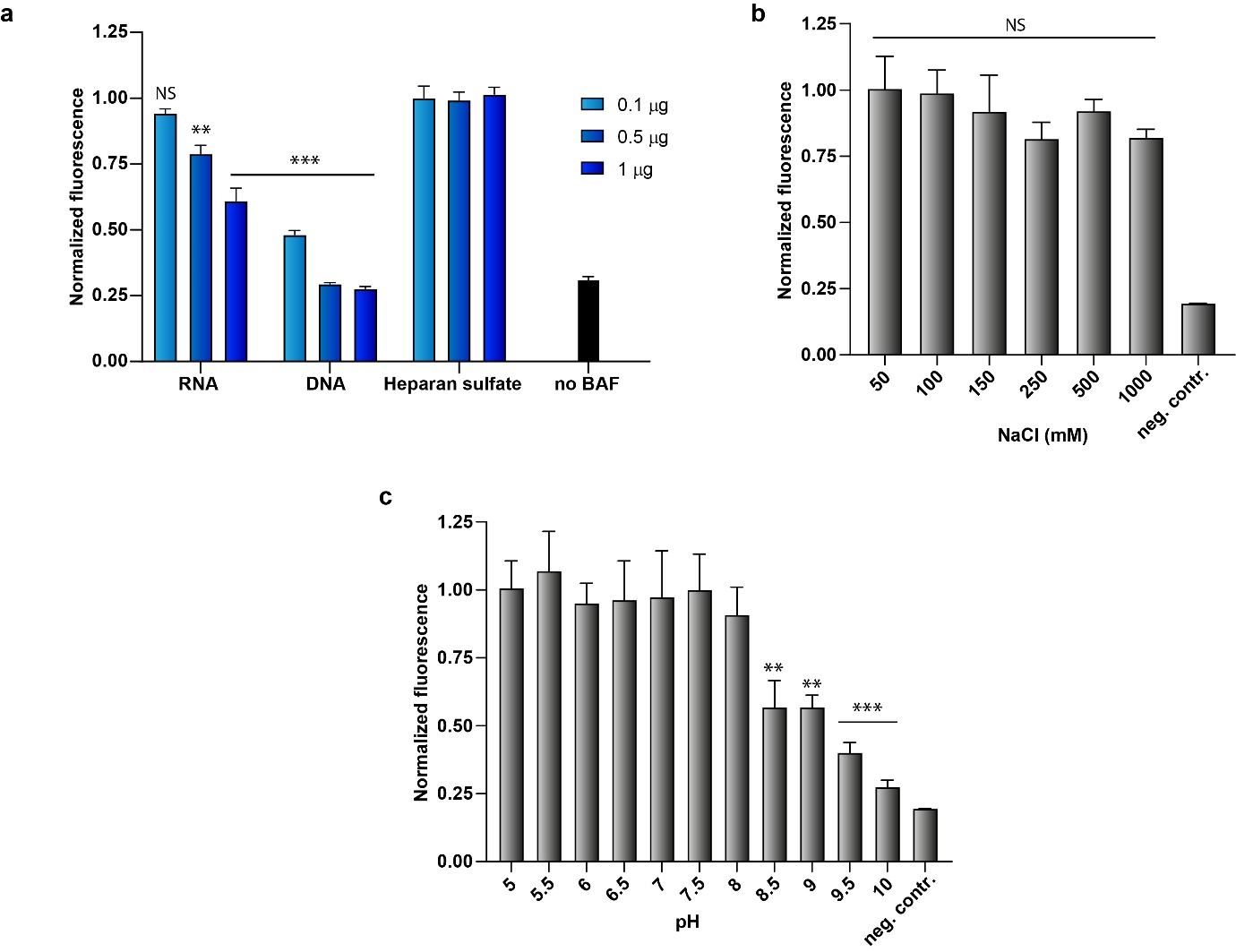


**Figure S3.** The HTS assay was performed using BAF (200 nM): **a)** in the presence of the indicated amount of RNA, unlabelled plasmid DNA and heparan sulfate per well, **b)** in varying NaCl concentrations and **c)** in a range of different pH conditions. All data were normalized and statistically compared to the BAF (200 nM) positive control under standard conditions in PBS. In the negative control no BAF was added. Mean + SD (N=3-4). ** p<0.01, *** p<0.01, NS=not significant respective to positive control.

**
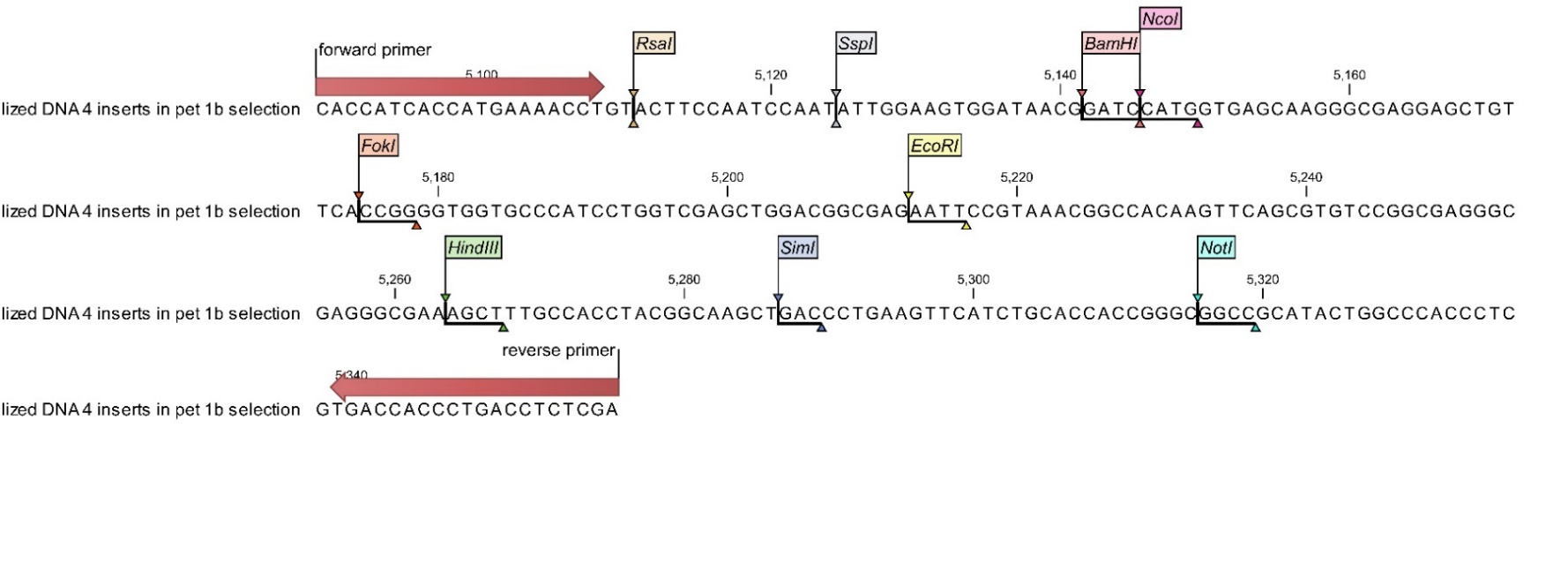
**

**Figure S4.** The 270 bp PCR product that was used for the HTS and the DNA fragment retention assay. The restriction enzyme cleavage sites and the amplification primer sequences (red arrows) are indicated.

**
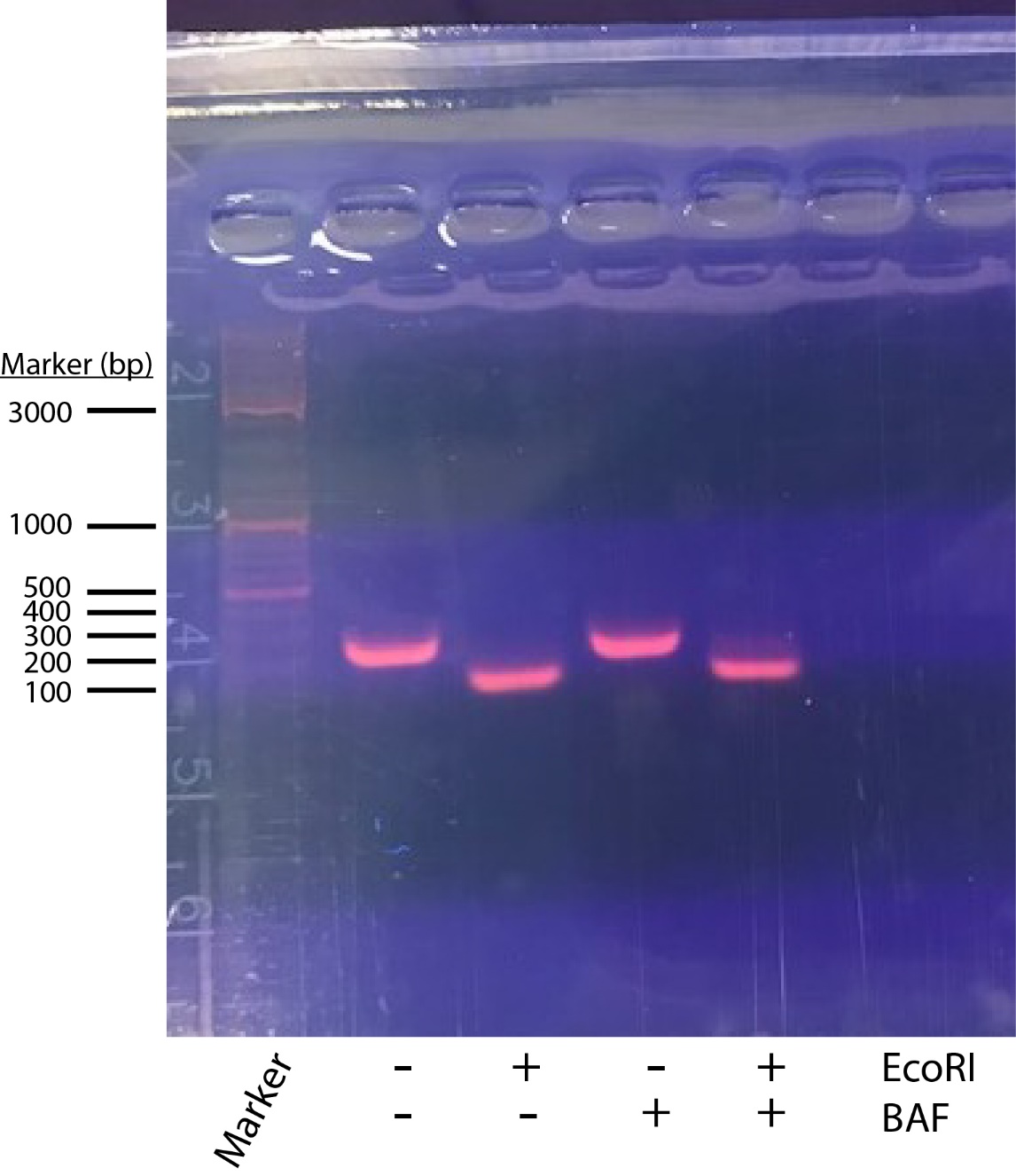
**

**Figure S5.** Restriction enzyme cleavage of DNA in the presence of BAF. The agarose gel shows the 270 bp PCR fragment with a central EcoRI cleavage site, which was incubated with EcoRI in the presence or absence of BAF (200 nM). A band shift is observed if EcoRI cleavage is successful. Cleary, the hydrolysis also occurs in the presence of BAF. After incubation with the restriction enzyme, SDS (1%, final concentration) was added to the samples, followed by incubation at 98°C for 5 min to inactivate BAF. Then, DNA loading buffer was added and samples were loaded on a agarose gel (1%).

**
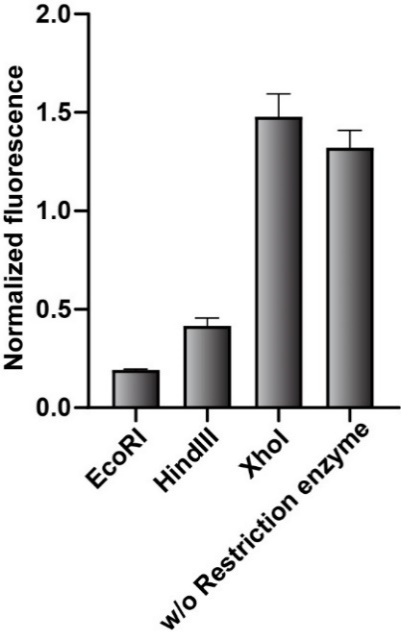
**

**Figure S6.** The DNA fragment retention assay was performed in the absence of BAF but in the presence of various DNA restriction enzymes. The fluorescence signal was normalized to the BAF (200 nM) positive control containing the respective restriction enzyme. The immobilized 270 bp DNA fragment contains one EcoRI, one HindIII, but no XhoI, cleavage sites. As expected, the fluorescently labelled DNA terminus is only cleaved off by EcoRI or HindIII. Mean + SD (N=3).


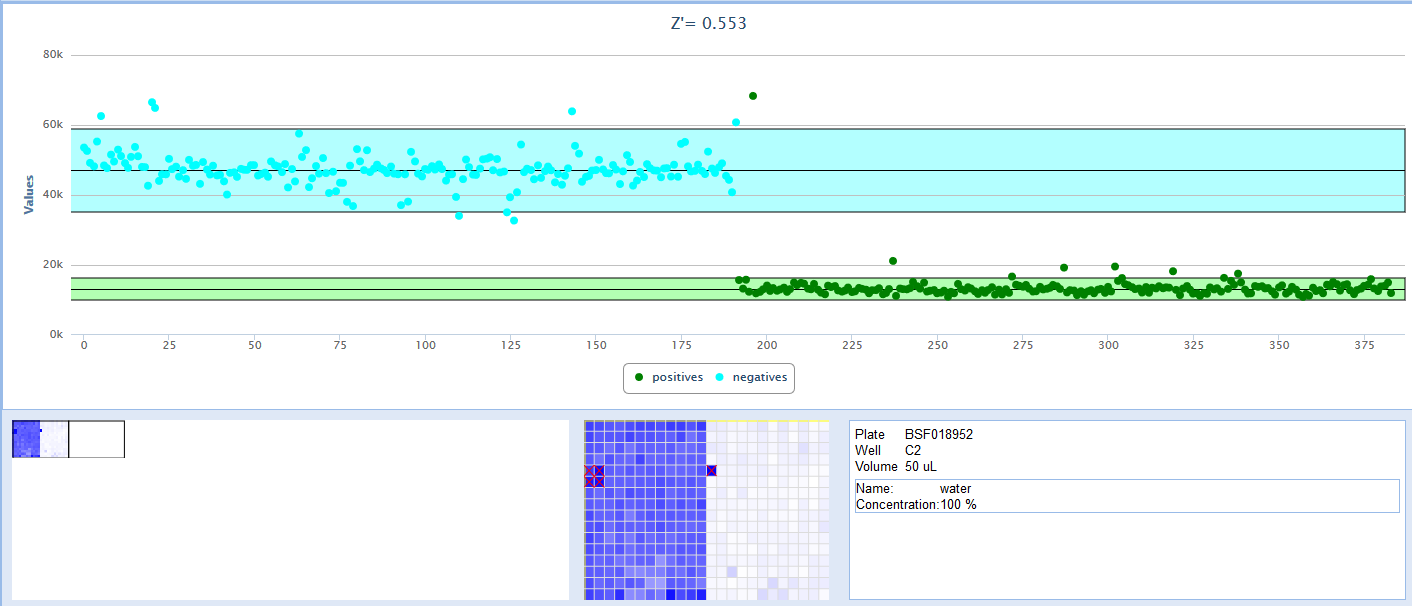


**Figure S7**. The Z’ factor of the automatized HTS was determined by loading half the wells of a 384 well plate with positive controls and half the plate with negative controls (no BAF added). The resulting fluorescence values (arbitrary units) are shown in blue (positive controls) and green (negative controls). The measurement was performed 3 h after the final PBS washing step. The 3xSD range is indicated for positive and negative controls by the blue and green areas, respectively. The well number is provided on the x-axis. The values of 5 outlayer wells have not been considered for statistical analysis, due to errors in the washing steps. These are indicated by a red cross on the schematic assay plate. The Z’ factor resulting from this plate was determined to be 0.553.


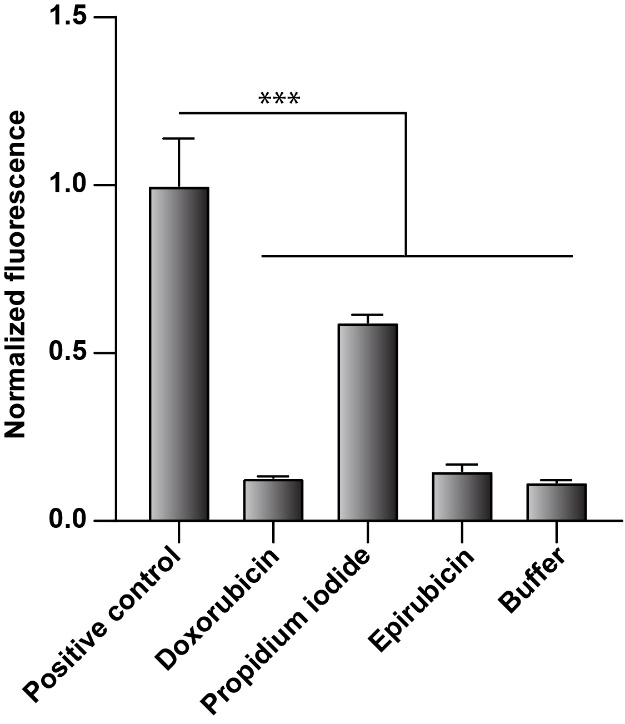


**Figure S8**. The quenching effect of DNA intercalators on ATTO 425-labelled DNA. Biotinylated and ATTO 425-labelled DNA was immobilized on a streptavidin coated multi-well plate. Subsequently, 10 µM of the indicated compounds were added. After 30 min of incubation at room temperature, the wells were thoroughly washed and ATTO 425 fluorescence was measured. All the tested DNA intercalators resulted in a significant quenching compared to the positive control where no compound was added. Mean + SD, normalized in regard to the positive control. *** p<0.01.

*ATCTCGATCCCGCGAAATTAATACGACTCACTATAGGGGAATTGTGAGCGGATAACAATTCCCCTCTAGAAATAATTTTGTTTAACTTTAAGAAGGAGATATACCATGGGTAGCAGCCATCATCATCATCACCACCAAGCTTCCGGAGGATCCGAAAATCTGTATTTCCAGATGACCACCAGTCAGAAACATCGTGATTTTGTTGCAGAACCGATGGGTGAAAAACCGGTTGGTAGCCTGGCAGGTATTGGTGAAGTTCTGGGTAAAAAACTGGAAGAACGCGGTTTTGATAAAGCCTATGTTGTTCTGGGTCAGTTTCTGGTGCTGAAAAAAGATGAGGACCTGTTTCGTGAATGGCTGAAAGATACCTGTGGTGCAAATGCAAAACAGAGCCGTGATTGTTTTGGTTGTCTGCGTGAATGGTGTGATGCATTTCTGTAAggCGAATTCCGGcgcactcgagcac*

**Figure S9.** TEV-BAF DNA sequence (by GeneArt Gene Synthesis). The BAF G27E mutant was cloned with the identical insert sequence. Except the GGT codon (blue) was changed to GAG to mutate glycine at position 27 to glutamic acid.


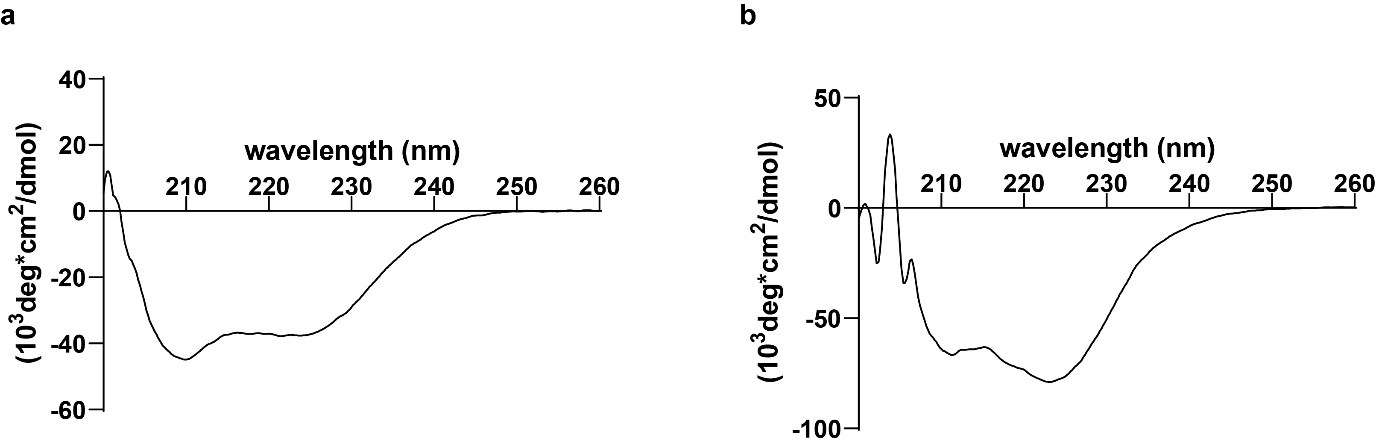


**Figure S10**. CD spectra of **a)** BAF (0.15 mg/mL) and **b)** MBP-LEM (0.4 mg/mL) in PBS.

ATCGGATCCGAAAACCTGTACTTCCAAAtggacaactacgcTgatctttcggataccgagctgaccaccttgctgcgccggtacaacatcccgcacgggcctgtagtaggatcaactcgtaggctttacgagaagaagatattcgagtacgagacccagaggAGACgatcGAATTCttaaGCTCGAGCAC

**Figure S11.** TEV-LEM (aa 1-47) DNA sequence (by GeneArt Gene Synthesis)

**Supplementary References**

1 Bradley, C. M., Ronning, D. R., Ghirlando, R., Craigie, R. & Dyda, F. Structural basis for DNA bridging by barrier-to-autointegration factor. *Nat Struct Mol Biol* **12**, 935-936, doi:10.1038/nsmb989 (2005).
